# Relation of Structural and Functional Changes in Auditory and Visual Pathways after Temporal Lobe Epilepsy Surgery

**DOI:** 10.3390/bs8100092

**Published:** 2018-10-12

**Authors:** Margarita Minou Báez-Martín, Lilia Maria Morales-Chacón, Iván García-Maeso, Bárbara Estupiñán-Díaz, María Eugenia García-Navarro, Yamila Pérez Téllez, Lourdes Lorigados-Pedre, Nelson Quintanal-Cordero, Ricardo Valdés-Llerena, Judith González González, Randis Garbey-Fernández, Ivette Cabrera-Abreu, Celia Alarcón-Calaña, Juan E. Bender del Busto, Rafael Rodríguez Rojas, Karla Batista García-Ramó, Reinaldo Galvizu Sánchez

**Affiliations:** 1 Epilepsy Surgery Program, International Center for Neurological Restoration, 25th Ave, No 15805, PC 11300 Havana, Cuba; lily@neuro.ciren.cu (L.M.M.-C.); ivangarciamaeso@gmail.com (I.G.-M.); baby@neuro.ciren.cu (B.E.-D.); marugeniagarcian@gmail.com (M.E.G.-N.); yamilaperez86@yahoo.es (Y.P.T.); lourdesl@neuro.ciren.cu (L.L.-P.); nquintanal@neuro.ciren.cu (N.Q.-C.); rvaldes@neuro.ciren.cu (R.V.-L.); judith@neuro.ciren.cu (J.G.G.); randis0770@gmail.com (R.G.-F.); ivettec@neuro.ciren.cu (I.C.-A.); calarcon@neuro.ciren.cu (C.A.-C.); jebender@infomed.sld.cu (J.E.B.d.B.); 2 Imaging Department, International Center for Neurological Restoration, 25th Ave, No 15805, PC 11300 Havana, Cuba; rrguezrojas@gmail.com (R.R.R.); kbatista@neuro.ciren.cu (K.B.G.-R.); 3 Clinical Department, International Center for Neurological Restoration, 25th Ave, No 15805, PC 11300 Havana, Cuba; rgalvizu@infomed.sld.cu

**Keywords:** auditory evoked responses, connectivity, drug-resistant epilepsy, temporal lobectomy, tractography, visual evoked potentials

## Abstract

Auditory and visual pathways may be affected as a consequence of temporal lobe epilepsy surgery because of their anatomical relationships with this structure. The purpose of this paper is to correlate the results of the auditory and visual evoked responses with the parameters of tractography of the visual pathway, and with the state of connectivity between respective thalamic nuclei and primary cortices in both systems after the surgical resection of the epileptogenic zone in drug-resistant epileptic patients. Tractography of visual pathway and anatomical connectivity of auditory and visual thalamus-cortical radiations were evaluated in a sample of eight patients. In general, there was a positive relationship of middle latency response (MLR) latency and length of resection, while a negative correlation was found between MLR latency and the anatomical connection strength and anatomical connection probability of the auditory radiations. In the visual pathway, significant differences between sides were found with respect to the number and length of tracts, which was lower in the operated one. Anatomical connectivity variables and perimetry (visual field defect index) were particularly correlated with the latency of P100 wave which was obtained by quadrant stimulation. These results demonstrate an indirect functional modification of the auditory pathway and a direct traumatic lesion of the visual pathway after anterior temporal lobectomy in patients with drug resistant epilepsy.

## 1. Introduction

The natural history of mesial temporal lobe epilepsy (mTLE) is variable; among 20% and 40% of patients have pharmacologically intractable seizures despite more than ten new antiepileptic drugs being added to the market in recent years [1,2,3,4]. This refractoriness to medication leads to a progressive deterioration from the neurobiological, psychological and social point of view of the patients. Hence, surgical resection of the epileptogenic zone continues to be an important and effective therapeutic option for people with mTLE, with seizures totally disappearing in around 70–85% of cases [3,5,6]. However, due to the morbidity of the procedure, the risk-benefit balance, as well as secondary effects, should be considered. Particularly, the surgery of the temporal lobe may involve structures related to sensory processing. From the anatomical and functional point of view, the temporal lobe is closely related to the auditory and visual pathways before its projection to the temporal operculum and the calcarine sulcus, respectively [7]. Thus, these pathways can be directly or indirectly affected as a consequence of surgical treatment.

Visual field defects may appear as a consequence of direct injury of the optic radiations during the surgical procedure (superior quadrantanopia contralateral to the side of resection), particularly the Meyer’s loop which runs in the vicinity of the temporo-mesial structures [8,9,10,11,12,13]. However, less is known about possible modifications that may occur in the auditory pathway [6,14].

In previous papers, we studied the variations of auditory and visual evoked potentials in patients with temporal lobe epilepsy that were submitted to surgical resection of the epileptogenic area [15,16]. Auditory brainstem response, auditory middle latency response and partial field visual evoked potentials were evaluated in particular. Additionally, some anatomical characteristics of the resected tissue and its relationship with the auditory and visual pathways were evaluated in a sample of these patients. A high coincidence rate between quadrant visual evoked potentials and perimetry was found, particularly in the superior quadrant that was contralateral to surgery. The volumetric study also showed that the injury of structures were probably related to the visual field defect. However, the results of the auditory evoked potentials and their relation to the volumetric findings pointed towards indirect damage of the auditory pathway, secondary to surgery. The aim of this paper is to correlate the results of the quadrant visual evoked potentials with the parameters of tractography of the visual pathway, and the results of the auditory and visual evoked responses with the state of connectivity between respective thalamic nuclei and primary cortices in both systems after the surgical resection of the epileptogenic zone.

## 2. Materials and Methods

### 2.1. Subjects

The study sample consisted of twenty-seven patients who were evaluated at the Telemetric Unit of the International Center for Neurological Restoration in Havana, Cuba in a prospective study. These patients were referred to this Unit from specialized consultations throughout the country once their intractability to medications was defined. They had crisis for at least two years with the use of at least two major antiepileptic drugs (Carbamazepine, Diphenylhydantoin, Valproate, Phenobarbital, Primidone) at the maximum tolerated doses for adequate periods of time. The inclusion criteria were:

- Patients with mTLE evaluated according to the institution’s protocols and who were candidates for resective surgery according to agreed criteria.
- Patients who met the drug-resistance criteria.
- Patients who gave their informed consent to participate in the investigation.

Presurgical evaluation was performed, including the localization of the ictal zone by combining Electroencephalogram-video, ictal and interictal SPECT (Single Photon Emission Computed Tomography), qualitative and quantitative MRI and neuropsychological tests. Clinical and demographical data of the patients and controls are shown in Table 1.

The most frequently used drugs for treatment before and after surgical procedure were carbamazepine, clonazepam and valproate, and this did not change for at least two years after the surgical procedure. By the time the study began, 88.8% of patients had taken more than one medication.

All patients underwent standard anterior temporal lobectomy guided by electrocorticography. Tissue samples that were obtained during surgery were histopathologically studied. Also, focal cortical dysplasias were classified according to Blumcke’s criteria [17] (Table 1).

Refraction defects were tested and corrected in every subject before the evaluation of the visual system. Additionally, hearing threshold level was evaluated.

All subjects were right-handed and gave their consent to participate in the study.

### 2.2. Electrophysiological Tests

Auditory brainstem response (ABR), middle latency response (MLR) and partial field visual evoked potentials were measured in all of the patients and healthy subjects.

The recording conditions of the auditory and visual electrophysiological tests are summarized in Table 2.

The records were obtained with a Neuropack four-mini and a Neuropack M1 device (Nihon Khoden, Tokyo, Japan).

The auditory stimuli were 0.1 ms alternating clicks that were delivered through a headphone (DR-531B-7, Elegas Acous Co. Ltd., Tokyo, Japan).

The visual stimuli consisted of a pattern-reversal checkerboard (black and white checks) with a fixation center-point, 16’ size and high contrast. They were presented on a VD-401A monitor.

### 2.3. Resection Measure

The absolute length of the lateral and mesial resected tissue was measured by the neurosurgeon using slices of MRI in T1, T2 and FLAIR (Fluid-Attenuated Inversion Recovery) six months after surgery. The lateral (neocortical) aspect included the distance (in mm) between the posterior edge of the internal table and the anterior limit of the resected area, considering the middle temporal gyrus in the axial slices of the MRI, whereas the mesial aspect was calculated from axial slices in parallel with the preserved hippocampus. Two tangential lines were drawn: one between the posterior border of the resection and the contralateral hippocampus, and the other between the anterior limit of the preserved hippocampus and the side of resection. The distance between these tangential lines was the mesial longitude.

### 2.4. Perimetry

The requirements for the perimetric evaluation can be reviewed in Báez Martín et al. 2010 (16). Moreover, the area of the superior quadrant that was ipsilateral to the resection was used as a reference of the subject itself [18], and the calculation of the visual field defect index (VFD-I) was carried out using the following expression:

VFD-I = 1 − (preserved area in the contralateral superior quadrants/preserved area in the ipsilateral superior quadrants), so that a high index corresponded to a greater visual defect.

### 2.5. Diffusion Tensor Imaging (DTI)

The diffusion neuroimaging studies were performed in eight patients, six with right lobectomy and two with left. By using the standard gradient direction scheme (twelve weighted diffusion images and one b = 0 image), the diffusion images were acquired by an echo-planar sequence (EPI) with the following parameters: b = 1200 s/mm^2^; FOV = 256 × 256 mm^2^; acquisition matrix = 128 × 128 mm^2^; corresponding to a spatial resolution 2 × 2 mm^2^; TE/TR = 160 ms/7000 ms.

For the processing of the images, the diffusion spectrum imaging (DSI) Studio software (dsi-studio.labsolver.org/dsi-studio-download) was used and the DTI method that was proposed by Basser et al. which characterizes the main direction of fiber diffusion in the brain [19].

### 2.6. Tractography

Quantitative measurements, such as fractional anisotropy (FA), apparent diffusion coefficient (ADC) as well as axial and radial diffusivity values were obtained. These measures were analyzed through predetermined areas, according to regions of interest (ROIs) that were positioned following anatomical guides. In the case of optic radiation, the ROI was drawn on the FA map of each subject individually, also evaluating the occipital cortex (area 17 of Brodmann) as an independent ROI. This analysis was not possible for the auditory pathway due to the poor visualization of the thalamus-cortical segment, which did not allow drawing the ROI on the FA map.

### 2.7. Brain Anatomical Connectivity

The condition of the connections between the thalamus and the cortex for the visual and auditory pathways was evaluated. In the case of the visual pathway, anatomical connectivity was measured between the lateral geniculate body and the primary visual cortex (area 17 of Brodmann). For the auditory pathway, the same analysis was performed between the medial geniculate body and the primary auditory cortex (area 41 of Brodmann). A methodology that was based on graph theory that was proposed by Iturria et al. (2007) was implemented to describe the specific connections between the different regions of gray matter. The method consists of three essential steps: (1) definition of a Brain Graph model in which each voxel is considered as a node of a non-directed weighted graph; (2) use of an iterative algorithm to find the route of maximum probability between two nodes and the subsequent definition of the anatomical connectivity measure between them; (3) definition of three anatomical connectivity measures between different gray matter regions: anatomical connection strength (ACS), anatomical connection probability (ACP) and anatomical connection density (ACD) [20].

### 2.8. Statistics

Data analysis included the comparison between hemispheres for tractography and connectivity six months after surgery (*t* test for dependent samples). Normalization of the electrophysiological data was carried out using the mean and standard deviation values of the control group for correlation studies. The relationship among electrophysiological tests, perimetry, length of resection, tractography and anatomical connectivity was measured by the Pearson correlation test. Differences were considered significant if *p* < 0.05 for all tests (Statistica 8.0.360 Copyright StatSoft. Inc., Tulsa, OK, USA, 1984–2011).

### 2.9. Ethical Considerations

All the procedures followed the rules of the Declaration of Helsinki of 1975 for human research, and the study was approved by the scientific and ethics committee from the International Center for Neurological Restoration (CIREN37/2012).

## 3. Results

### 3.1. Auditory Pathway

As already noted, it was not possible to evaluate the tracts corresponding to the auditory radiations, however it was possible to study their connections. The results (mean and standard deviation) of the variables that were considered for this study are summarized in Table 3.

#### Structure-Function Relationships

For the correlation analysis with the anatomical variables, using the normalized data, both groups of patients were united, considering whether the pathway to be evaluated was ipsi or contralateral to the resection.

*ABR:* There were no statistically significant correlation between the variables of the ABR and the length of resection (Pearson correlation test, *p* > 0.05). There was a negative correlation between the connection density and the latency of the wave III that was ipsilateral to the resection (Pearson correlation test, *p* < 0.05, r = −0.76) only in the analysis of the connectivity in the auditory radiations. This showed a significant prolongation of latency in the group with right lobectomy.

*MLR:* As a whole, we found a positive relationship between the latency of the MLR components and the length of the resection; whereas a negative one was observed with the connectivity of the auditory thalamus-cortical radiation.

Table 4 summarizes the results of the correlation analysis between the electrophysiological variables of the MLR and the anatomical studies (Pearson correlation test, *p* < 0.05).

The latency of Na correlated positively with the length of neocortical resection, however only in the group of patients with right lobectomy (Pearson correlation test, *p* < 0.05, r = 0.61). This group is precisely the one that showed a prolongation of latency of this component after resection, which reached statistical significance twelve months after surgery (*t* test, *p* < 0.05). It was also the group that showed the most extensive resection in the temporal neocortex (average 42.81 mm). The latency of the components Pa and Nb correlated positively with the mesial length of the resection in the whole group of operated patients (Pearson correlation test, *p* < 0.05, r = 0.51 and r = 0.48, respectively).

In a previous report, it described the positive correlation between the amplitude of Na and Pa waves and the resected volume in the middle temporal pole (Pearson correlation test *p* < 0.05, r = 0.84 and r = 0.65, respectively), especially in left temporal lobectomy patients. On the contrary, negative correlations were previously observed between the amplitude of these components and the residual indexes of the middle temporal pole (Pearson correlation test, *p* < 0.05, Na: r = −0.89 y Pa: r = −0.79), inferior temporal gyrus and amygdala (Pearson correlation test, *p* < 0.05, only Pa: r = −0.67 y r = −0.59, respectively) [15].

### 3.2. Visual Pathway

The results (mean and standard deviation) of the variables that were considered for the white matter tracts of optic radiation in both hemispheres are summarized in Table 5.

The comparative analysis of the tracts that were related to the occipital cortex showed differences between the sides that were statistically significant for the number of tracts, volume of tracts and fractional anisotropy (*t* test for dependent samples), which was also lower on the operated side.

Table 6 shows the results (mean and standard deviation) of the variables that were considered in the study of anatomical connectivity. The comparisons between the sides showed lower values of density, probability and strength of the anatomical connection on the side of the resection. However, these differences did not reach statistical significance (*t* test for dependent samples, *p* > 0.05). 

#### Structure-Function Relationships

For the analysis of correlation with the anatomical studies, both groups of patients were united considering whether the quadrant to be evaluated was ipsi or contralateral to the resection, and standardized data of the quadrant-visual evoked potentials was used. The longest latency (maximum latency) and the smallest amplitude (minimum amplitude) of the P100 wave were taken from the pair of affected quadrants.

Table 7 summarizes the results of the correlation analysis that had statistical significance among the electrophysiological variables, the perimetry and the anatomical studies (Pearson correlation test, *p* < 0.05).

**Figure 1:**
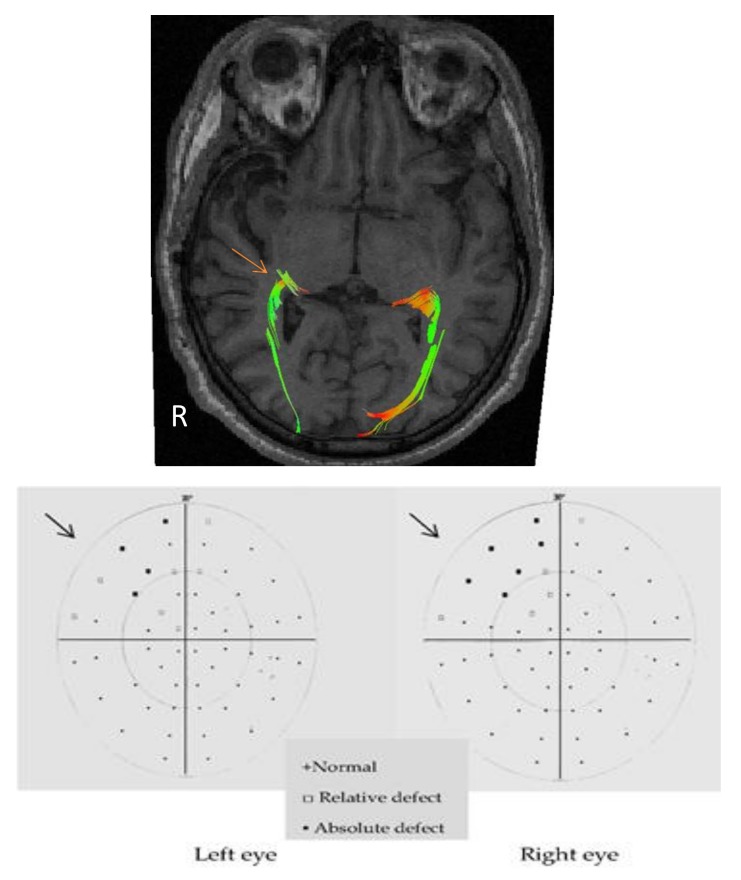
Tractography of optic radiations and perimetry of a patient with right lobectomy. Arrows indicate the pathological optic radiation (tractography) and the affected quadrants (perimetry). R: right side of the image.

Figure 1 shows an example of the tractography of optic radiation in a patient with right lobectomy, where the reduction in the number of fibers on that side is appreciated and whose perimetry showed a superior left quadrantanopia.

## 4. Discussion

Epilepsy surgery as a therapeutic option for patients with drug-resistant mTLE produced structural and functional changes in the auditory and visual sensory systems, which were detected by the use of electrophysiological techniques and in correspondence with the structural variations that were evidenced with neuroimaging techniques.

The relationships that were found between the brainstem exploration (ABR) on the same side of the resection and the density of the connection in the auditory pathway could correspond to the known fact that the fibers which participate in the efferent control that are exerted by the cortex on the lower levels of the auditory pathway (mesencephalon-pons-cochlear receptor) are mostly ipsilateral [21].

It is known that the primary auditory cortex, preserved during this surgery, inhibits the conduction of the ascending impulses by means of the sensory control system, modifying the activity in the relay stations of the pathway (inferior colliculi and olive-cochlear complex) ipsilaterally, while the stimulation of secondary auditory areas enhances the ascending impulses [21]. These latter areas could be influenced by the removed zone, especially in patients with right lobectomy where the resection is wider. As a result of this excitatory-inhibitory imbalance, the inhibition of the aference on the same side of the surgery dominates, which is expressed by a delay in conduction at the subcortical levels of the pathway.

The magnitude of the resection of mesial structures, which is greater in the group with left temporal lobectomy, had greater repercussions on the thalamus-cortical components of the MLR (Pa, Nb), while the extension of the resection in its neocortical lateral aspect exerted a greater influence on the most caudal component (Na), especially in the group with right lobectomy. All these results speak in favor of a possible functional relationship between the resected structures and the generators of these components, with a variable behavior depending on whether the operated hemisphere is right or left.

The study of the anatomical connectivity showed an inverse relationship of the probability and strength of connection (particularly its FA) with the latency of Pa and Nb for the auditory radiation of the operated hemisphere. Interestingly, the Na component was related to the connection probability: the higher the latency and the lower the amplitude, the lower the connection probability (see Table 4).

These results suggest the existence of a modification of the functional state in the auditory pathway, which is given by a lower speed of propagation of the nerve impulse in correspondence with a reduction in the flow of information from the medial geniculate body to the auditory cortex and a lower functional relationship among these structures, with repercussion to other lower levels in the neuroaxis (inferior colliculi-superior olive complex).

However, if we take into account that during the postsurgical evaluation, the amplitude analysis of the components of the MLR that were far from decreasing showed a tendency to increase at 12 and 24 months after the resection [15], we could be in the presence of a “paradoxical” phenomenon of release, characterized by a long-term functional improvement once the epileptogenic zone is removed.

The possibility that this change is dependent on a physical factor, such as the reduction of impedance at the surgical site, does not seem feasible, given that the increase in amplitude was progressive over time.

The results of the auditory examination confirm what has been proposed in previous studies about the occurrence of long-term neuroplastic changes secondary to an indirect effect of the resection of temporal lobe structures [15]. Although our data seem to point to a possible effect of the surgery in the auditory pathway, these measures are not fully independent. In summary, there are functional implications of mTLE surgery on auditory processing. Concerning the visual pathway, the damage of Meyer’s loop after temporal lobectomy [9,10,22,23,24,25,26,27,28,29,30] is well known, with the consequent visual field defect (homonymous superior quadrantanopia contralateral to the resected side) that can limit activities of daily life like driving [28,31].

Unlike the postulates that were used in the evaluation of the auditory pathway, in the case of the visual pathway, the sequelae of the anterior temporal lobectomy do constitute, a consequence of the direct aggression of the optical radiation fibers, dependent on the surgical procedure employed [32] and the distance between the tip of the Meyer loop and the posterior edge of the temporal pole [33]. Its anatomical disposition predisposes for this type of affectation to occur, especially in patients with right lobectomy where there is no risk of damaging eloquent areas for language, with which more generous removals from the epileptogenic zone can be carried out guided by trans-operative electrocorticography.

However, none of these studies have considered the relationship between functional variations and anatomical changes of the visual pathway.

The latency of the P100 wave that was obtained with quadrant stimulation correlated with the fiber damage in optic radiation, which was demonstrated by the effect of the extension of the resection in the neocortex of the temporal lobe (positive) and the value of the connectivity between the lateral geniculate body and the occipital cortex (negative) on this electrophysiological variable. This relationship was also evident between the latency of the P100 wave and the results of the perimetry (positive) for the three recording electrodes.

The amplitude of P100 correlated with the volume of the tracts that were related to the occipital cortex. The density and strength of the connection between the regions of interest had a positive relationship with this variable, selectively for the O2 electrode (right occipital cortex), which seems to be congruent given that most of the patients that were included in the anatomical study of the tracts had resection of the anterior temporal lobe of the right side (6 of 8). Furthermore, subjects with the higher volume of resected tissue showed the most delayed latencies and the lowest P100 amplitude [16].

Note that perimetry, classically considered the “gold standard” for the evaluation of visual field defects, also had a statistically significant relationship with the neocortical length of the resection and with the values of fractional anisotropy, both from the optic radiation and the occipital cortex (See Table 7).

When evaluating the tractography variables in optic radiation, we found no statistically significant relationship with the remaining measurements in the studied subjects (Pearson correlation test, *p* > 0.05).

However, when considering the side of the resection and the responses of each eye separately, a negative correlation of the P100 latency in the contralateral superior quadrant of the ipsilateral eye was found with the values of fractional anisotropy, and a positive correlation with the ADC and diffusivity (axial and radial) in patients with right lobectomy. This result suggests, as it did with visual potential and perimetry, that there is a higher probability of damaging fibers from the eye on the same side of the resection that derive from the temporal retina (nasal visual field) and that run more ventrally in the Meyer’s loop, causing the incongruous visual field defects [8]. 

Finally, it is worth noting that we found a statistically significant negative correlation of the tract volume and the fractional anisotropy of the occipital cortex of the operated side with the P100 latency in the contralateral superior quadrant (Pearson correlation test, *p* < 0.05, r = −0.87 and r = −0.89 respectively), and with the amplitude of P100 in the same quadrant (Pearson’s correlation test, *p* < 0.05, r = 0.94 and r = 0.92, respectively). The above was valid only when the analysis of the patients with right temporal lobectomy was performed. Once again, the results point to a greater anatomical and functional compromise of optic radiation when it comes to the non-dominant hemisphere for language.

In summary, the highest values of P100 latency in the contralateral superior quadrant indicate probable myelin damage in the optic radiation, which is supported by neuroimaging studies of diffusion (tractography and connectivity) and in correspondence with the perimetric results [16]. All of these findings confirm the existence of a partial and selective dysfunction of the visual pathway, in particular of the optic radiations on the side of the temporal lobectomy, which is expressed as a superior homonymous quadrantanopia contralateral to the resection, which is imperceptible for most patients with epilepsy that are resistant to medication and subjected to this surgical procedure.

These visual sequels could be minimized with transoperative monitoring guided by tractography of optic radiations, a method that is not available today in all groups that perform this type of surgery.

Among the limitations of this study is the absence of presurgical references of the magnetic resonance images in both pathways, as well as the difficulties to evaluate the images of the auditory radiations.

## 5. Conclusions

Taken as a whole, these results demonstrate an indirect functional modification of the auditory pathway and a direct traumatic lesion of the visual pathway after anterior temporal lobectomy in patients with pharmaco-resistant epilepsy.

The novelty of our study lies in the combined use of electrophysiological and imaging techniques to evaluate sensory pathways that are related to resected tissue in patients undergoing this surgical procedure. Undoubtedly, the results of tractography and structural connectivity are in line with the findings of the functional tests, which further strengthens our study.

## Figures and Tables

**Table 1:** Clinical and demographical characteristics of the sample.

		N	Sex		Age Years μ (SD)	Duration of Illness Years μ (SD)	Temporo-Mesial Sclerosis N	Focal Cortical Dysplasia		
			M	F				IIIa	IIIb	No
**Patients**										
	**Left** lobectomy	14	5	9	33.07 (9.06)	21.14 (9.08)	14	8	2	4
	**Right** lobectomy	13	7	6	34.69 (5.28)	22.53 (12.14)	13	9	1	3
**Healthy subjects**		16	8	8	33.68 (7.96)	-	-	-		

μ: mean value; SD: standard deviation.

**Table 2:** Recording conditions of auditory responses and visual evoked potentials.

Parameters	ABR	MLR	VEP
Analysis time (ms)	10	100	300
Filters (Hz)	100–3000	20–1000	1–100
Stimulus frequency (Hz)	10	5	1
Maximal intensity (dBnHL)	105	90	-
Average responses	2000	500	30
Sensibility (μV/div)	5	20	20
Recording electrodes	A1, A2	Cz	O1, Oz, O2
Reference electrode	Cz	A1-A2	Fz
Ground electrode	Fpz	Fpz	A1
Stimulation mode	Monoaural	Binaural	Quadrants

ABR: auditory brainstem response; MLR: middle latency response; VEP: visual evoked potentials.

**Table 3:** Auditory radiations variables of DTI (Diffusion Tensor Imaging).

Variables	Ipsilateral Auditory Radiation		Contralateral Auditory Radiation		
	µ	SD	µ	SD	*p*
Density of connection	0.193	0.0773	0.251	0.0660	0.026 *
Density of connection (FA)	0.068	0.0293	0.093	0.0189	0.0800
Density of connection (MD)	0.080	0.0306	0.112	0.0261	0.0652
Anatomical Connection probability	0.422	0.1613	0.498	0.1172	0.3981
Anatomical Connection probability (FA)	0.181	0.0992	0.210	0.0372	0.4783
Anatomical Connection probability (MD)	0.193	0.0965	0.230	0.0565	0.4317
Anatomical connection strength	726.900	402.7487	1214.176	330.9955	0.0346 *
Anatomical connection strength (FA)	257.677	149.3024	449.342	100.6329	0.020 *
Anatomical connection strength (MD)	302.172	161.8940	542.212	135.5526	0.0214 *

FA: fractional anisotropy MD: media diffusivity. µ: media; SD: standard deviation. * Statistically significant differences between sides (*t* test for dependent samples, *p* < 0.05).

**Table 4:** Correlations between middle latency response and anatomical variables.

	Na Latency	Pa Latency	Nb Latency	Na Amplitude
Mesial length of resection	+0.13	+0.51 *	+0.48 *	−0.39
Neocortical length of resection **	+0.61 *	+0.31	+0.21	+0.16
Anatomical connection probability (FA) **	−0.88 *	−0.86	−0.89 *	+0.92 *
Anatomical connection strength (FA) *	−0.87	−0.89 *	−0.89 *	0.83

Note: The table refers to the correlation of the anatomical variables that were ipsilateral to the resection with the normalized values of middle latency response recorded in Cz. The + and − signs indicate whether the relationship is positive or negative, and the numbers correspond to the r of each correlation (Pearson correlation test, *p* < 0.05). FA: fractional anisotropy. ** Patients with right temporal lobectomy. * Statistically significant values.

**Table 5:** DTI values in optic radiation and occipital cortex. Tractography.

		Ipsilateral		Contralateral		
		µ	SD	µ	SD	*p*
OR	Number of tracts **	356.625	185.997	578.500	155.902	0.0041 *
	Length of tracts	19.671	7.139	26.870	7.557	0.0307 *
	Volume of tracts	3167.500	1339.976	4368.750	1462.917	0.1988
	FA	0.403	0.127	0.499	0.031	0.0587
	ADC	0.001	0.000	0.001	0.000	0.1448
	AD	0.001	0.000	0.001	0.000	0.3502
	RD	0.001	0.000	0.001	0.000	0.1228
OC	Number of tracts	1961.87	832.04	2971.25	661.71	0.0017 *
	Length of tracts	14.986	4.748	18.190	2.669	0.1127
	Volume of tracts	7396.25	3425.97	9145.00	2655.12	0.0499 *
	FA	0.348	0.083	0.388	0.099	0.0026 *
	ADC	0.001	0.000	0.001	0.000	0.1062
	AD	0.001	0.000	0.001	0.000	0.7131
	RD	0.001	0.000	0.001	0.000	0.0741

OR: optic radiation; OC: occipital cortex. FA: fractional anisotropy; ADC: apparent diffusion coefficient; AD: axial diffusivity; RD: radial diffusivity. μ: average; SD: standard deviation. ** Right lobectomy patients. (*t* test for dependent samples, *p* < 0.05). * Statistically significant differences between sides. When comparing the optic radiations of both sides, statistically significant differences were evidenced for the whole group of patients with respect to the length of the tracts (*t* test for dependent samples, *p* = 0.0307) being smaller on the operated side. When we limited the analysis to the group with right lobectomy, the number of tracts was also significantly different (*t* test for dependent samples, *p* = 0.0041).

**Table 6:** DTI values in optic radiation. Anatomical connectivity.

	Ipsilateral		Contralateral		
	µ	SD	µ	SD	*p*
Anatomical connection density	0.155	0.0733	0.174	0.0757	0.6104
Anatomical connection density (FA)	0.050	0.0271	0.069	0.0323	0.2115
Anatomical connection density (MD)	0.059	0.0306	0.075	0.0305	0.3426
Anatomical connection probability	0.387	0.1810	0.404	0.1292	0.8029
Anatomical connection probability (FA)	0.134	0.0711	0.174	0.0512	0.2581
Anatomical connection probability (MD)	0.178	0.0885	0.181	0.0537	0.9418
Anatomical connection strength	890.987	450.569	1239.440	610.7670	0.2174
Anatomical connection strength (FA)	290.970	171.595	487.988	260.4703	0.0958
Anatomical connection strength (MD)	342.933	188.255	531.343	253.9019	0.1243

FA: fractional anisotropy; MD: medium diffusivity. μ: average; SD: standard deviation (*t* test for dependent samples, *p* > 0.05).

**Table 7:** Correlations with statistical significance between the anatomical variables, quadrant visual evoked potentials and the perimetry.

**(A) Optic Radiations**	**P100 Latency**			**P100 Amplitude**			**Perimetry (VFD-I)**
**Electrodes**	**O1**	**Oz**	**O2**	**O1**	**Oz**	**O2**	
Perimetry	+0.53	+0.50	+0.50				
Neocortical length of resection	+0.61	+0.60	+0.59				+0.72
FA *	−0.96	−0.98	−0.97				−0.73
ADC *	+0.98	+0.97	+0.98				
Axial diffusivity *	+0.99	+0.99	+0.99				
Radial diffusivity *	+0.99	+0.99	+0.99				
Connection density	−0.79	−0.78	−0.78			+0.76	
Connection probability	−0.89	−0.87	−0.86				
Connection strength	−0.88	−0.88	−0.89			+0.74	
**(B) Occipital Cortex**	**P100 Latency**			**P100 Amplitude**			**Perimetry (VFD-I)**
**Electrodes**	**O1**	**Oz**	**O2**	**O1**	**Oz**	**O2**	
Number of tracts						+0.86	
Volume of tracts				+0.87	+0.94	+0.80	
FA					+0.82		−0.71

Note: The table refers to the correlation of the anatomical variables with the normalized values of the visual evoked potential by quadrants (maximum latency and minimum amplitude) in the contralateral superior quadrant to the resection. The + and − signs indicate whether the relationship is positive or negative, and the numbers correspond to the r of each correlation (Pearson correlation test, *p* < 0.05). * Patients with right lobectomy, right eye. VFD-I: visual field defect index. FA: fractional anisotropy; ADC: apparent diffusion coefficient.
